# Comparison of coronary atherosclerotic disease burden between ST‐elevation myocardial infarction and non‐ST‐elevation myocardial infarction: Non‐culprit Gensini score and non‐culprit SYNTAX score

**DOI:** 10.1002/clc.23534

**Published:** 2020-12-28

**Authors:** Takamasa Tanaka, Kojiro Miki, Hirokuni Akahori, Takahiro Imanaka, Nagataka Yoshihara, Toshio Kimura, Koji Yanaka, Masanori Asakura, Masaharu Ishihara

**Affiliations:** ^1^ Department of Cardiovascular and Renal Medicine Hyogo College of Medicine Hyogo Japan

**Keywords:** coronary atherosclerotic disease burden, Gensini score, non‐ST‐elevation myocardial infarction, ST‐elevation myocardial infarction, SYNTAX score

## Abstract

**Background:**

Patients with non‐ST‐elevation myocardial infarction (NSTEMI) have worse long‐term prognoses than those with ST‐elevation myocardial infarction (STEMI).

**Hypothesis:**

It may be attributable to more extended coronary atherosclerotic disease burden in patients with NSTEMI.

**Methods:**

This study consisted of consecutive 231 patients who underwent coronary intervention for myocardial infarction (MI). To assess the extent and severity of atherosclerotic disease burden of non‐culprit coronary arteries, two scoring systems (Gensini score and synergy between percutaneous coronary intervention with Taxus and cardiac surgery [SYNTAX] score) were modified by subtracting the score of the culprit lesion: the non‐culprit Gensini score and the non‐culprit SYNTAX score.

**Results:**

Patients with NSTEMI had more multi‐vessel disease, initial thrombolysis in myocardial infarction (TIMI) flow grade 2/3, and final TIMI flow grade 3 than those with STEMI. As compared to STEMI, patients with NSTEMI had significantly higher non‐culprit Gensini score (16.3 ± 19.8 vs. 31.2 ± 25.4, *p* < 0.001) and non‐culprit SYNTAX score (5.8 ± 7.0 vs. 11.1 ± 9.7, *p* < 0.001).

**Conclusions:**

Patients with NSTEMI had more advanced coronary atherosclerotic disease burden including non‐obstruction lesions, which may at least in part explain higher incidence of cardiovascular events in these patients.

## 1 INTRODUCTION

It has been well demonstrated that patients with non‐ST‐elevation myocardial infarction (NSTEMI) have worse long‐term prognosis than those with ST‐elevation MI (STEMI).^1^, ^2^ Although it may be in part explained by higher age, more previous MI, more comorbid factors, and more multi‐vessel disease in patients with NSTEMI, NSTEMI is independently associated with worse prognosis even after adjustment of these factors. Occlusive multi‐vessel disease is defined as the presence of significant stenosis in the non‐culprit artery. However, extent of non‐obstructive coronary atherosclerotic disease burden is also the important determinant of patient outcome, and may be attributable to the poor prognosis of patients with NSTEMI. There are no reports that compared of the extend of coronary atherosclerotic disease burden including non‐obstructive lesions between STEMI and NSTEMI in angiographic scoring applications.

Several coronary scoring systems have been proposed to assess the extent of atherosclerotic disease burden including both obstructive and non‐obstructive lesions: the Gensini score^3^ and the synergy between percutaneous coronary intervention with Taxus and cardiac surgery (SYNTAX) score.^4^ In the current study, we modified them by subtracting the score of the culprit lesion of MI: “non‐culprit Gensini score” and “non‐culprit SYNTAX score”. Using these two modified scoring systems, we compared the extend of coronary atherosclerotic disease burden including non‐obstructive lesions between patients with STEMI and those with NSTEMI.

## 2 METHODS

**FIGURE 1:**
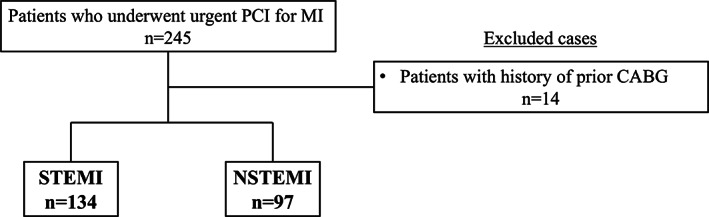
Study flow diagram. CABG, coronary artery bypass grafting; MI, myocardial infarction; NSTEMI, non‐ST‐elevation myocardial infarction; PCI; percutaneous coronary intervention; STEMI; ST‐elevation myocardial infarction

From April 2015 to November 2018, a total of 245 consecutive patients who hospitalized within 48 h of the onset of acute myocardial infarction (AMI) followed by underwent urgent percutaneous coronary intervention (PCI) at Hyogo College of Medicine (Hyogo, Japan) were retrospectively reviewed. Of those, 14 patients with a prior history of coronary artery bypass grafting (CABG) were excluded. The 231 patients who underwent urgent PCI were analyzed (Figure 1). This protocol was based on the accordance with the Declaration of Helsinki. The protocol was approved by the ethical committee of Hyogo College of Medicine (IRB no 3347) and registered to the University Hospital Medical Information Network Clinical Trials Registry (UMIN‐CTR) (ID: UMIN000038372).

STEMI was diagnosed in the new presence of ST‐elevation at the J‐point at least two contiguous leads or new left bundle branch block in 12 or 15 leads electrocardiogram (ECG). NSTEMI was diagnosed by raise or fall of cardiac troponin and absence of persistent ST elevation in ECG. The culprit lesion was determined by the interpretation of the clinical patient's status, cardiac biomarker, ECG assessment, and angiographic findings.

The Gensini score is a comprehensive score that assesses the extent of coronary atherosclerotic disease burden on angiography. This score was calculated the sum of severity score assigned depending on the degree of angiographic luminal stenosis each segment in the coronary artery: Geometrically increasing severity of lesions–25%, 50%, 75%, 90%, 99%, and 100% coronary stenosis, cumulative effect of multiple lesions, and lesion location. Lesions with stenosis 25% or more are targets for its evaluation.

The SYNTAX score was developed during the SYNTAX trial to provide the information regarding complexity of coronary artery disease (CAD).^4^ It is calculated in each individual coronary artery lesion that has a diameter stenosis greater than 50%, evaluating with dominance of artery distribution, coronary segment, presence of total occlusion, trifurcation or bifurcation or not, aorta ostial lesion, tortuosity, lesion length, calcification, and thrombus. It is calculated with the internet SYNTAX calculator (version 2.28).

To evaluate the coronary atherosclerotic disease burden of the non‐culprit lesions, these two scoring applications were modified as “non‐culprit Gensini score” and “non‐culprit SYNTAX score”, which were calculated by subtracting the point of the culprit lesion from the original score. Because some patients have a total occlusion (Thrombolysis in Myocardial Infarction (TIMI) flow grade 0 or 1) in the culprit lesion, the scoring was performed after reperfusion so that distal lesions could be counted. To assess the reliability of quantitative angiographic scoring measurements, these scores were measured by two dedicated interventional cardiologists blinded to the clinical information. Additionally, the scores on a total of randomly selected 30 patients were re‐evaluated by a single observer to identify intra‐observer variability, at two separate time points. From measurements conducted by two independent observers, the inter‐observer variability was confirmed.

Continuous variables were presented as the mean ± SD unless otherwise noted, and compared using the unpaired Student's *t* test (for parametric data) and the Mann–Whitney U test (for non‐parametric data). Categorical variables were expressed as percentages, and assessed using the chi‐squared test or Fisher's exact test. The reliability of continuous variables in angiographic scores was provided as the intra‐class correlation coefficient. All statistical tests were two‐sided and *P* values<0.05 were regarded as significant. Statistical analyses were performed using JMP version 13 (SAS Institute, Cary, NC).

## 3 RESULTS

The patient and lesion characteristics are shown in Tables 1, 2. There were 134 patients with STEMI and 97 patients with NSTEMI. The mean age was significantly younger in the STEMI patients than the NSTEMI patients. Similarly, prevalence of dyslipidemia and prior PCI was more in patients with NSTEMI compared with those with STEMI. On the other hand, peak Creatine Phosphokinase and Killip class was more severe in patients with STEMI. Initial angiography found a total occlusion (TIMI flow grade 0–1) of the culprit lesion more frequently in the STEMI patients (63% vs. 15%, *p* < 0.05). Reperfusion with TIMI flow grade 2–3 was obtained in all patients.

**TABLE 1:** Patients characteristics

	STEMI	NSTEMI	*p* value
	(*N* = 134)	(*N* = 97)	
Age (years)	69 ± 12	74 ± 10	<0.05
Male	104 (78)	74 (76)	0.81
Coronary risk factor			
Hypertension	96 (73)	78 (80)	0.18
Diabetes	54 (41)	47 (48)	0.28
Dyslipidemia	54 (40)	55 (57)	<0.05
Current Smoker	68 (52)	47 (49)	0.58
Family history	15 (11)	6 (6)	0.19
eGFR (<45 ml/min/1.73m^2^)	34 (26)	27 (28)	0.66
Previous history			
PAD	12 (10)	10 (11)	0.80
Stroke	13 (6)	9 (4)	0.91
MI	18 (14)	20 (21)	0.15
Previous PCI	24 (18)	32 (33)	<0.05
Systolic blood pressure (mmHg)	130.6 ± 44.2	134.3 ± 26.4	0.47
Heart rate (beats/min)	78.4 ± 27.7	77.8 ± 18.8	0.85
GRACE score	117.4 ± 31.3	119.7 ± 26.2	0.56
Max CK (IU/L)	2876 ± 3047	1080 ± 1939	<0.05
Killip class (≥2)	46 (39)	14 (17)	<0.05

*Note*: Values are mean ± SD or number (%).

Abbreviations: CK, creatine kinase, eGFR, estimated glomerular filtration rate; GRACE, global registry of acute coronary events; LVEF, left ventricular ejection fraction, MI, myocardial infarction; PAD, peripheral arterial disease, PCI, percutaneous coronary intervention.

**TABLE 2:** Lesion characteristics

	STEMI	NSTEMI	*p* value
	(*n* = 134)	(*n* = 97)	
Culprit vessel			
RCA	49 (37)	37 (38)	0.08
LAD	72 (54)	40 (41)	
LCX	10 (7)	17 (18)	
LMT	3 (3)	3 (4)	
Multi‐vessel disease	32 (41)	47 (59)	<0.05
Stent used	118 (88)	87 (90)	0.34
Initial TIMI 0 flow	84 (63)	14 (15)	<0.05
Final TMI 3 flow	103 (79)	90 (96)	<0.05

*Note*: Values are mean ± SD or number (%).

Abbreviations: LAD, left anterior descending coronary artery; LCX, left circumflex coronary artery, LMT: left main trunk; RCA, right coronary artery; TIMI, thrombolysis in myocardial infarction.

**FIGURE 2:**
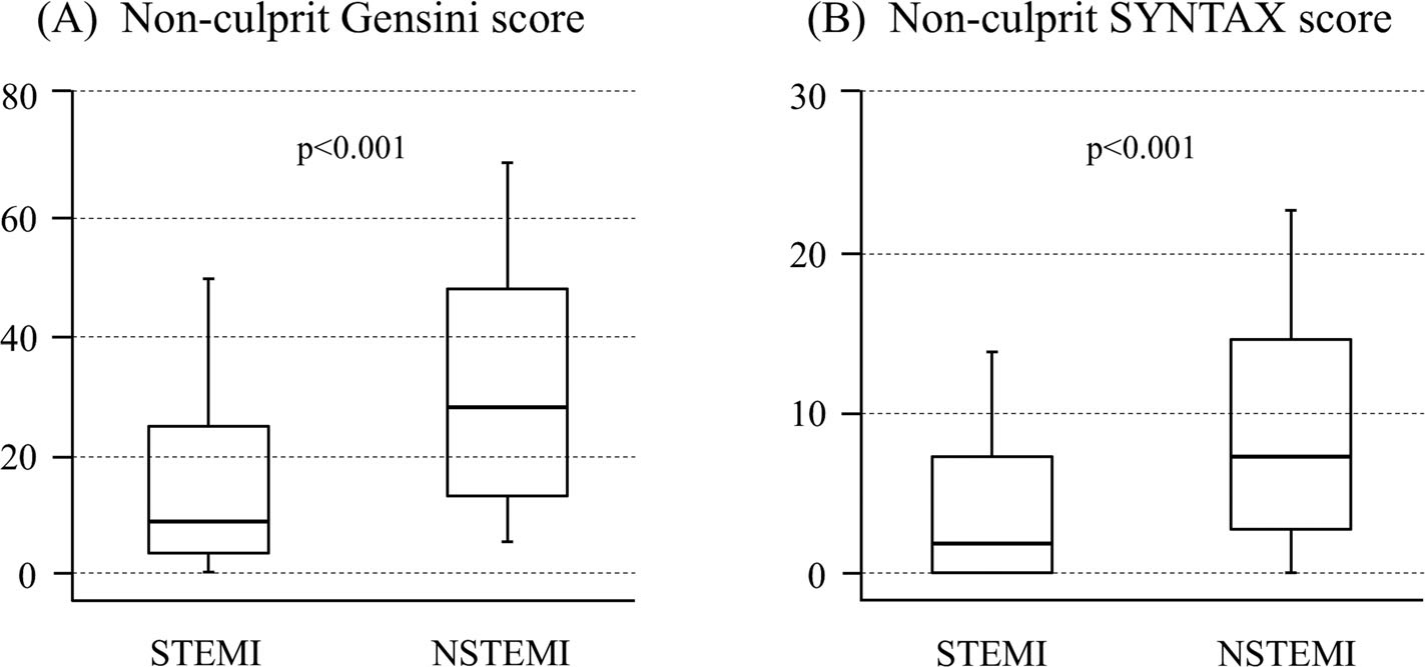
Box‐and‐whisker diagram. Comparison of angiographic extent of coronary atherosclerotic disease burden in non‐culprit lesion between STEMI and NSTEMI. (A) The non‐culprit Gensini score and (B) the non‐culprit SYNTAX score. STEMI; ST‐elevation myocardial infarction; NSTEMI, non‐ST‐elevation myocardial infarction

Figure 2 shows the non‐culprit Gensini score and the non‐culprit SYNTAX score. The non‐culprit Gensini score was 16.3 ± 19.8 in‐patient with STEMI. In‐patient with NSTEMI, the non‐culprit Gensini score was 31.2 ± 25.4, which was nearly two fold higher as compared to those with STEMI (*P* < 0.001). Similarly, as compared to patient with STEMI, the non‐culprit SYNTAX score was significantly higher in patients with NSTEMI (5.8 ± 7.0 vs. 11.1 ± 9.7, *p* < 0.001).

The comparison of the non‐culprit Gensini score and the non‐culprit SYNTAX score between STEMI and NSTEMI stratified by age and presence of dyslipidemia, diabetes, and prior PCI are presented in Table 3. The non‐culprit Gensini score and the non‐culprit SYNTAX score were significantly higher in patients with NSTEMI compared with those with STEMI regardless of the patient characteristics.

**TABLE 3:** Comparison of the non‐culprit Gensini score and non‐culprit SYNTAX score between STEMI and NSTEMI stratified by baseline characteristics

	Non‐culprit Gensini score			Non‐culprit SYNTAX score		
	STEMI	NSTEMI	*P* value	STEMI	NSTEMI	*P* value
≥ 75y						
Yes	18.8 ± 22.9	34.8 ± 28.1	0.003	7.0 ± 8.4	12.7 ± 11.1	0.005
No	14.6 ± 17.4	28.1 ± 22.7	<0.001	4.9 ± 5.8	9.6 ± 8.3	<0.001
Dyslipidemia						
Yes	19.9 ± 22.9	32.2 ± 24.8	0.009	6.3 ± 8.1	11.3 ± 9.5	0.004
No	13.8 ± 17.1	29.8 ± 26.3	<0.001	5.4 ± 6.2	10.5 ± 10.2	<0.001
Diabetes						
Yes	19.6 ± 20.1	37.2 ± 27.0	<0.001	7.4 ± 7.1	13.8 ± 10.3	<0.001
No	14.5 ± 19.6	25.5 ± 22.5	0.005	4.7 ± 6.8	8.4 ± 8.5	0.011
Prior PCI						
Yes	22.4 ± 24.4	37.8 ± 26.0	0.028	7.0 ± 9.3	13.7 ± 10.8	0.019
No	14.9 ± 18.5	27.9 ± 24.5	<0.001	5.4 ± 6.4	9.6 ± 8.9	<0.001

*Note*: Values are mean ± SD.

Abbreviations: NSTEMI, non‐ST‐elevation myocardial infarction; PCI, percutaneous coronary intervention; STEMI, ST‐elevation myocardial infarction; SYNTAX, synergy between percutaneous coronary intervention with Taxus and cardiac surgery.

Angiographic scores exhibited excellent intra‐observer and inter‐observer reliability. Intra‐class correlation coefficients for intra‐observer and inter‐observer variability of the non‐culprit Gensini score were respectively 0.987 and 0.994. In addition, the non‐culprit SYNTAX score also showed excellent points (0.991 and 0.962) for intra‐observer and inter‐observer reliability.

## 4 DISCUSSION

Previous studies have reported that the long‐term incidence of the cardiovascular events was higher in patients with NSTEMI than in those with STEMI.^1^, ^2^ Patients with NSTEMI have more comorbid risk factors such as elderly age, hypertension, diabetes and dyslipidemia, which may in part account for poor prognosis of these patients. However, even after adjustment of these clinical factors, patient with NSTEMI is independently related with poor prognosis. Atherosclerotic disease burden is an important determinant of prognosis. A previous study has shown that coronary atherosclerotic disease burden in patient with non‐Q wave MI is more extended than those with Q‐wave MI.^5^ The majority of patients with ST elevation develop a Q‐wave MI and those without ST elevation develop a non‐Q‐wave MI, whereas, the minority develop a non‐Q‐wave MI and Q‐wave MI respectively.^6^ It is not clear whether the extent of coronary atherosclerotic disease burden in patients with NSTEMI is more extended compared those with STEMI in the coronary scoring applications.

In the current study, we compared the extent of coronary atherosclerosis might be different between STEMI and NSTEMI. To assess the extent of coronary atherosclerotic disease burden of non‐culprit lesions, including both obstructive and non‐obstructive lesions, we proposed the non‐culprit Gensini score and the non‐culprit SYNTAX score, which were calculated by subtracting points of the culprit lesion from the original Gensini score and SYNTAX score.

We showed that the non‐culprit Gensini score and the non‐culprit SYNTAX score were significantly higher in patients with NSTEMI than in those with STEMI. These findings suggest that patients with NSTEMI have more extended coronary atherosclerotic disease burden, which may be responsible for poor prognosis of these patients.

Coronary atherosclerotic disease burden is the important determinant of prognosis in patients with coronary artery disease. AMI is precipitated by coronary thrombosis subsequent to plaque rupture or erosion, which may occur not only at obstructive but also non‐obstructive coronary lesion.^7^, ^8^, ^9^ Pathological and angiographic observations have shown that most atherosclerotic plaques responsible for acute MI are mild to moderate, non‐obstructive lesions. In studies using coronary calcium scanning and stress test in the same patients, coronary calcium score was associated with increased risk of subsequent cardiac events despite normal stress test results.^10^, ^11^ Studies using coronary computed tomography angiography have also reported the association of non‐obstructive coronary artery disease and subsequent cardiac events. These findings indicate the importance of coronary atherosclerotic disease burden at non‐obstructive as well as obstructive lesions for the risk assessment of subsequent cardiac events.

The Gensini score is comprehensive score that assesses the extent and severity of coronary atherosclerotic disease burden on the angiography. This scoring application calculates including mild to moderate stenosis (diameter stenosis >0%).^3^ It has been reported that the Gensini score is well correlated with atherosclerotic plaque burden assessed by intravascular ultrasound.^12^ Therefore, the Gensini score is appropriate scoring applications used to assess the coronary artery atherosclerosis disease burden in several studies. The non‐culprit Gensini score is novel applications to quantify the extent and severity of coronary artery atherosclerotic disease burden in the non‐culprit lesions, subtracting the culprit lesion from the original Gensini score. Because distal to an occluded culprit lesion was invisible, the scoring was performed on the angiograms after reperfusion.

It has been reported that the Gensini score correlates with other angiographic scoring systems that assess atherosclerotic disease burden, including SYNTAX score. The SYNTAX score is widely used as a clinically relevant score that predicts prognosis in patients undergoing PCI. The original SYNTAX score is calculated on the angiograms before PCI. The residual SYNTAX score, which is calculated on the angiograms after PCI, has been developed to assess the prognosis of patients who underwent multiple PCIs.^13^ In the current study, the purpose was to assess total atherosclerotic disease burden of non‐culprit lesions in patients with MI rather than link to subsequent clinical outcomes. Therefore, we used the non‐culprit Gensini score and the non‐culprit SYNTAX score, not residual score. PCI was limited only for the culprit lesion and no patient received PCI for the non‐culprit lesion during the index procedure.

Using the non‐culprit Gensini score and the non‐culprit SYNTAX score, we assessed coronary atherosclerotic disease burden in the non‐culprit lesions in patients with STEMI and those with NSTEMI. We showed that the non‐culprit Gensini score and the non‐culprit SYNTAX score were significantly higher in patients with NSTEMI than those with STEMI. These findings suggested that coronary atherosclerotic disease burden is more extended in‐patient with NSTEMI.

The present study was based on retrospective analysis of a relatively small number of patients from a single center. Multicenter, prospective studies with a larger number of patients would be required to reconfirm our results, using these non‐culprit Gensini score and non‐culprit SYNTAX score. It is remains unclear what extent of the totally atherosclerotic burden is causally related. It is needed to investigate the relationship between the prognosis and the non‐culprit scoring system in the future.

## 5 CONCLUSIONS

The non‐culprit Gensini score and the non‐culprit SYNTAX score provide useful information on the extent of atherosclerosis disease burden in patients with MI. Patients with NSTEMI had more extended coronary atherosclerosis disease burden in the non‐culprit lesions, which might be responsible for the poor prognosis in those patients.

## Data Availability

The data that support the findings of this study are available from the corresponding author, Masaharu Ishihara, upon reasonable request.
